# Association between the types of bystander cardiopulmonary resuscitation and the survival with good neurologic outcome of preschool pediatric out-of-hospital cardiac arrest cases in Japan: A propensity score matching analysis using an extended nationwide database

**DOI:** 10.3389/fped.2022.1075983

**Published:** 2023-02-01

**Authors:** Kenshi Murasaka, Akira Yamashita, Hitoshi Owada, Yukihiro Wato, Hideo Inaba

**Affiliations:** ^1^ Department of Emergency Medicine, Kanazawa Medical University, Uchinada, Japan; ^2^ Department of Cardiology, Noto General Hospital, Nanao, Japan; ^3^ Department of Emergency Medical Sciences, Niigata University of Health and Welfare, Niigata, Japan; ^4^ Kanazawa University, Kanazawa, Japan

**Keywords:** preschool pediatric out-of-hospital cardiac arrest, epidemiologic feature, bystander cardiopulmonary resuscitation, conventional cardiopulmonary resuscitation, compression-only cardiopulmonary resuscitation, neurologically favorable 1-month survival rate.

## Abstract

**Background:**

Pediatric out-of-hospital cardiac arrests (OHCAs) are frequently associated with a respiratory etiology. Despite the high proportion of preschool children with OHCAs, very few studies on this special population exist. This study characterizes the epidemiologic features of preschool pediatric OHCAs and analyzes the advantage of conventional (ventilations with chest compressions) bystander cardiopulmonary resuscitation (CPR) over compression-only bystander CPR (BCPR) on the one-month post-event neurological status of the patient.

**Methods:**

Japanese nationwide databases for all ambulance transport events and OHCAs occurring during a 4-year period between 2016 and 2019 were combined, totalling 3,608 patient events. Children ≤6-years-old were included; physician- and EMS-witnessed events, no prehospital resuscitation effort events, and neonatal patient events were excluded. Neurologically favorable 1-month survival rates were compared among groups using univariate and multivariate analyses before and after propensity score matching.

**Results:**

From the combined database, 2,882 pediatric OHCAs meeting selection criteria were categorized as no BCPR (984), compression-only BCPR (1,428), and conventional BCPR (470). The proportion of bystander-witnessed cases was low (22.3%). Most OHCA witnesses were family members (88.5%), and most OHCAs occurred at home (88.0%). The neurologically favorable 1-month survival rates were: no BCPR 2.4%, compression only, 3.2%, and conventional 6.6% (*P* < 0.01). Multivariate logistic regression analysis before and after matching showed that conventional BCPR was associated with higher neurologically favorable 1-month survival than compression-only BCPR. Subgroup analyses after matching demonstrated that conventional BCPR was associated with better outcomes in nonmedical (adjusted odds ratio; 95% confidence interval, 2.83; 1.09–7.32) and unwitnessed OHCA cases (3.42; 1.09–10.8).

**Conclusions:**

Conventional CPR is rarely performed by bystanders in preschool pediatric OHCA. However, conventional BCPR results in neurologically favorable outcomes in nonmedical and unwitnessed cases.

## Introduction

Cardiopulmonary resuscitation (CPR) guidelines recommend conventional CPR using ventilation combined with chest compressions for pediatric out-of-hospital cardiac arrests (OHCAs) (1–4), particularly in pediatric OHCAs of asphyxial etiology. Pediatric OHCAs are less likely to be cardiac in origin and are more frequently associated with a respiratory etiology than adult OHCAs (3). Sudden infant death syndrome (SIDS) is the leading cause of pediatric OHCAs (29.6%–59.5%), followed by airway obstruction, trauma, and submersion (5, 6).

In Japan and other countries, most pediatric OHCAs occur in preschool children (40.1%–69.8%), while infants account for 23.5%–47.4% (7–9) of pediatric OHCAs (7, 10). The incidence of infantile OHCAs is 75.3 per 100,000 infants per year, which is similar to adult OHCAs (11). The survival rate of pediatric in-hospital cardiac arrests have improved, but the survival rate of pediatric OHCAs remains poor (2, 12). For hospital admissions for OHCAs, the survival rates at discharge are 3.3% for infants and 9.1% for 1–11-year-old children (2). OHCAs in older children may occur during school and outdoor activities, but OHCAs in younger children are more likely to happen at home (7, 9). Therefore, family members are more likely to witness or detect OHCAs, and family CPR directly influences outcomes. Few reports have focused on preschool OHCAs, despite their high proportion among total pediatric OHCAs. Therefore, this study focuses on preschool OHCAs not witnessed by physicians or emergency medical services (EMS). The efficacy of conventional bystander CPR (BCPR) was compared with that of compression-only BCPR. We created a comprehensive OHCA database of preschool aged-patients by combining and reconciling a nationwide OHCA database with another database, including detailed information on locations, to investigate the relationship between characteristics of preschool OHCAs and the benefits of conventional BCPR.

## Methods

### Population and setting

In 2016, 5.6% of Japan's population of 126 million comprised preschool children aged ≤6 years (13). No termination of resuscitation rules exist for prehospital settings; unless a patient with an OHCA is clearly dead (such as decapitation) or exhibits postmortem changes, EMS personnel continue resuscitation until arrival at the hospital. Authorized paramedics are permitted to insert tracheal tubes and administer intravenous adrenaline (epinephrine); however, neither procedure is indicated for children aged ≤6 years (14). For OHCA cases, EMS personnel follow protocols founded by the regional medical control councils based on the Japan Resuscitation Council Guidelines (15). In this study, we defined the age of preschool children as ≤6 years old. In Japan, children who turn 6 years old start school in April. The data in this study did not include the date of birth and may therefore include a few school-aged children.

### Data selection

Consent was obtained from the Japanese Fire and Disaster Management Agency (FDMA) to analyze nationwide OHCA data prospectively collected between Jan 1, 2016, and Dec 31, 2019. The All-Japan Utstein Registry of FDMA contains Utstein-style data (16), including patient sex, age, witness status, initial electrocardiogram rhythm, prehospital defibrillation, prehospital physician involvement, adrenaline administration, advanced airway management, critical time recordings, any return of spontaneous circulation (ROSC), and survival at 1 month with cerebral performance category (CPC) (17). ROSC is determined by palpable carotid artery during rhythm check. To construct a comprehensive database, the FDMA registry data were combined and reconciled with another EMS transportation database that included detailed information on locations, time records, and in-hospital diagnoses.

### Ethics and patient consent

The requirement for patient informed consent was waived because the data were obtained from an anonymous database. This population-based observational study was approved by the Kanazawa Medical University Medical Research Ethics Review Committee (no. 1729).

### Outcome measures

The primary outcome was neurologically favorable 1-month (1-M) survival, defined as a CPC score of 1 (good recovery) or 2 (moderate disability) (17).

### Statistical analysis

The BCPR groups were classified as no BCPR, compression-only BCPR, and conventional (ventilations combined with chest compressions) BCPR. Epidemiological features, including witness status, sex, location (home, preschool-related location, and private and public bath), OHCA causes (medical, trauma including traffic accident, and submersion), shockable initial rhythm, and prehospital defibrillation, were compared among the BCPR groups. The medical causes of OHCAs included respiratory, cardiac, and cerebrovascular diseases. Differences in nominal variables were assessed using the chi-square test. Crude and adjusted odds ratios (ORs), using 95% confidence intervals (CIs), were calculated. The null hypothesis was evaluated for each analysis at a two-sided significance level of *P* < 0.05. Univariate analyses were performed to compare the epidemiology and characteristics of OHCAs among patients without BCPR, compression-only BCPR, and conventional BCPR. The neurologically favorable 1-M survival rate was compared using univariate and multivariate logistic regression analyses. The most critical comparisons between compression-only BCPR and conventional BCPR were repeated after propensity score matching of several influencing factors including season, weekday, sex, age, indication and implementation of prehospital defibrillation, cardiac/non-cardiac, medical/non-medical, location of occurrence, EMS response time, EMS transportation time, and classification of bystander (family/others). In the propensity score matching, the caliper value, which is the maximum permitted difference between matched subjects, was set at 0.05. Subgroup analyses based on medical, non-medical, witnessed, and unwitnessed OHCAs were performed before and after matching. All statistical analyses were performed using JMP Pro 16 software (SAS Institute, Cary, NC, USA).

## Results

### Case selection

**Figure 1:**
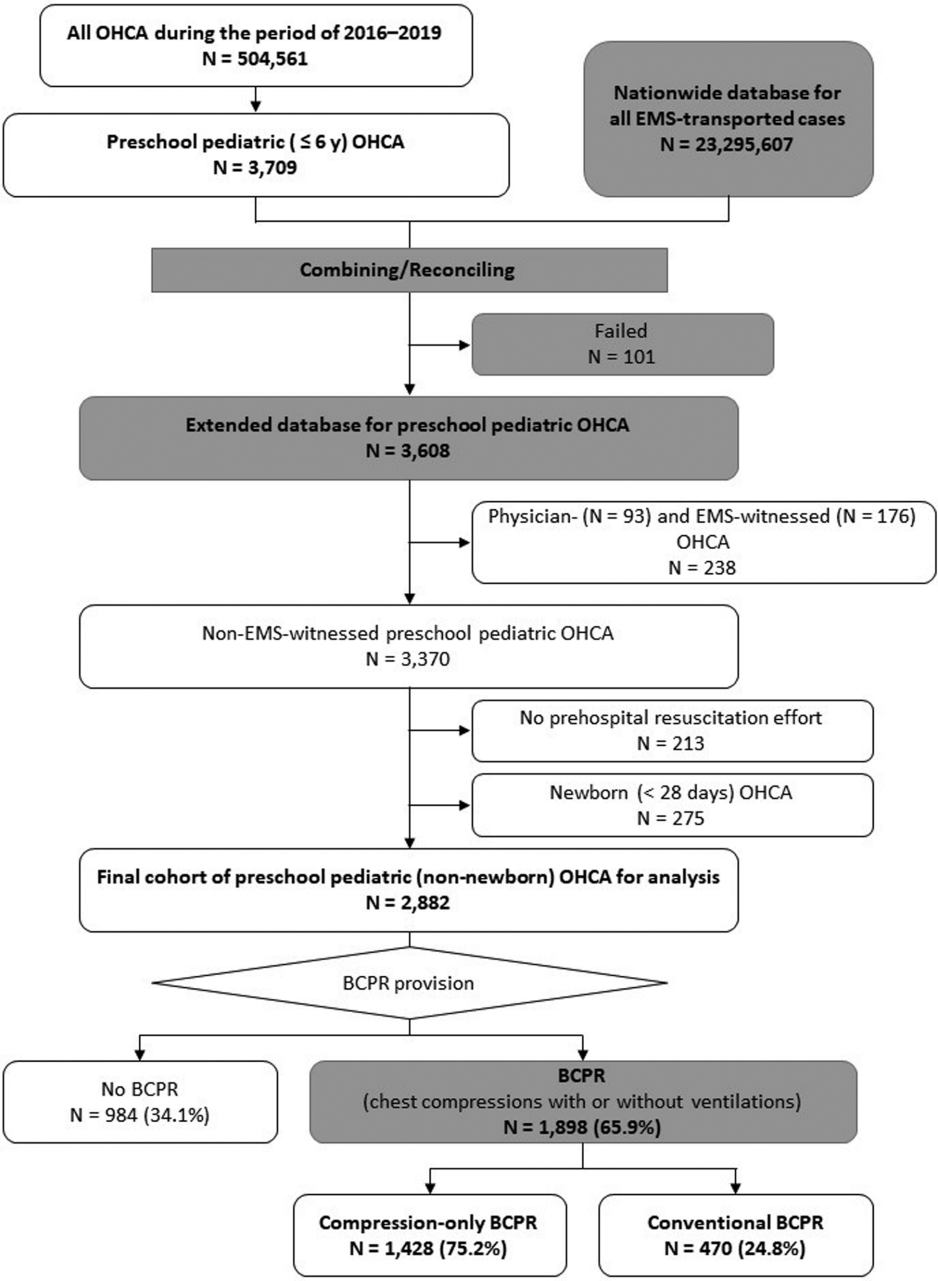
Case selection.

A comprehensive database of 3,608 preschool pediatric (≤6 years) OHCA cases during the study period were merged from the national database and the EMS transportation database. After excluding physician- and EMS-witnessed cases, no prehospital resuscitation effort cases, and neonatal (**<**28 days) cases, 2,882 cases were analyzed. BCPR was not performed in 984 cases (34.1%) and was implemented in 1,898 cases (65.9%), including 1,428 cases (75.2%) of compression-only BCPR and 470 cases (24.8%) of conventional BCPR (Figure 1).

### Characteristics and BCPR classification of preschool pediatric OHCAs

Of all the OHCAs, 22.3% (642/2,882) were witnessed by bystanders. The proportion of cases witnessed by bystanders was low regardless of the BCPR classification. Family members accounted for 88.5% (568/642) of the bystanders. No significant differences were detected in terms of patient sex. The proportion of infants <1 year of age was 57.6% (1,660/2,882). The majority of OHCAs (88.0%, 2,536/2,882) occurred at home, and 4.6% occurred in private and public baths and in pools. Conventional BCPR was performed at higher rates in preschool-related areas (childcare facilities, parks, and water places), baths, and pools. The cause of the OHCA was nonmedical in 25.3% (729/2882), of which 555 cases were traumatic. Submersion accounted for 6.5% (187/2882). The appearance of a shockable initial rhythm and any prehospital defibrillation performed before hospitalization were 2.6% and 2.3%, respectively. Any prehospital ROSC was observed in 168 cases and was more common in conventional BCPR (Table 1).

**Table 1:** Characteristics and locations of preschool pediatric OHCA cases.

OHCA characteristics and locations	All OHCA cases (*N* = 2,882)	Bystander CPR classification			
		No (*N* = 984)	Compression-only (*N* = 1,428)	Conventional (*N* = 470)	Chi-square or Fisher's exact probability test
Bystander-witnessed, % (*N*)	22.3 (642)	24.9 (245)	19.3 (276)	25.7 (121)	*P* < 0.01
Family-witnessed, % (*N*)	19.7 (568)	21.3 (210)	17.5 (250)	23.0 (108)	*P* = 0.01
Male, % (*N*)	58.3 (1,680)	57.9 (570)	57.6 (822)	61.3 (288)	*P* = 0.35
Age, % (*N*)	** **	** **	** **	** **	*P* < 0.01
0 years (excluding neonates)	57.6 (1660)	58.5 (576)	59.4 (848)	50.2 (236)	** **
1 years	17.7 (509)	15.1 (149)	18.7 (267)	19.8 (93)	** **
2 years	7.0 (201)	6.7 (66)	6.7 (96)	8.3 (39)	** **
3 years	5.6 (161)	6.7 (66)	4.1 (58)	7.9 (37)	** **
4 years	4.1 (117)	4.2 (41)	3.9 (56)	4.3 (20)	** **
5 years	4.1 (118)	4.0 (39)	4.1 (58)	4.5 (21)	** **
6 years	4.0 (116)	4.8 (47)	3.2 (45)	5.1 (24)	** **
Location, % (*N*)					
Home	88.0 (2,536)	85.4 (840)	90.1 (1,287)	87.0 (409)	*P* < 0.01
Preschool-related location^a^	4.4 (127)	4.1 (40)	3.6 (52)	7.5 (35)	*P* < 0.01
Childcare facilities	1.6 (46)	1.1 (11)	1.5 (21)	3.0 (14)	
Water places	1.5 (44)	1.8 (18)	0.9 (13)	2.8 (13)	
Amusement facilities, hotel, and park	1.3 (37)	1.1 (11)	1.3 (18)	1.7 (8)	
Private and public baths and pools	4.6 (132)	3.0 (29)	4.9 (70)	7.0 (33)	*P* < 0.01
OHCA causes, % (*N*)					
Non-medical causes	25.3 (729)	27.9 (275)	23.3 (333)	27.0 (121)	*P* = 0.04
Traumatic cases including traffic accident	19.3 (555)	19.5 (192)	18.8 (268)	20.2 (95)	*P* = 0.76
Submersion	6.5 (187)	4.7 (46)	6.7 (96)	9.6 (45)	*P* < 0.017
Shockable initial rhythm, % (*N*)	2.6 (74)	2.9 (28)	2.2 (31)	3.2 (15)	*P* = 0.35
Any prehospital defibrillation, % (*N*)	2.3 (66)	2.6 (26)	1.9 (27)	2.8 (13)	*P* = 0.32
Time factor, min, median (IQR)					
EMS response time interval	9 (7–11)	9 (7–11)	9 (7–11)	9 (8–11)	*P* = 0.67
EMS transportation time interval	11 (7–17)	11 (7–17)	12 (7–17)	11 (7–16)	*P* = 0.30
ROSC, % (*N*)	5.8 (168)	4.4 (43)	4.4 (43)	10.6 (50)	*P* < 0.01

^a^ Childcare facilities, seacoast, river, amusement facilities, and park.

### Outcomes among the bystander CPR groups before matching

In a preliminary analysis, any BCPR increased the survival rate compared to no BCPR (adjusted OR; 95% CI, 1.99; 1.23–3.23). Neurologically favorable 1-M survival was observed in 3.5% of cases (101/2,882). As shown in Table 2, the neurologically favorable 1-M survival rates were 2.4% (24/984), 3.2% (46/1,428), and 6.6% (31/470) in the no-BCPR, compression-only BCPR, and conventional BCPR groups, respectively (*P* < 0.01). In the multivariate logistic regression analysis of all OHCA cases, outcomes after compression-only BCPR were not significantly better than those after no BCPR (1.58; 0.94–2.67). However, the outcomes for conventional CPR were significantly better than those for no BCPR (3.23; 1.82–5.72) and compression-only CPR (2.04; 1.25–3.35). In considering why conventional CPR has an advantage over compression-only CPR, interactions were detected among OHCA causes (medical or nonmedical), witness status, and BCPR groups; thus, subgroup analyses were conducted. In the medical cause and witness OHCA subgroup analyses, both compression-only BCPR (2.64; 1.21–5.78 and 3.09; 1.51–6.33, respectively) and conventional BCPR (3.23; 1.31–7.94 and 3.63; 1.57–8.39, respectively) were significantly associated with better outcomes than no BCPR. However, conventional BCPR was not better than compression-only BCPR (1.22; 0.59–2.52 and 1.17; 0.59–2.34, respectively). In the nonmedical cause and unwitnessed OHCA subgroup analyses, only conventional BCPR (3.61; 1.68–7.75 and 2.68; 1.23–5.86, respectively) was significantly associated with better outcomes compared to no BCPR. Conventional BCPR was also better than compression-only BCPR (2.97; 1.49–5.94 and 3.43; 1.60–7.33, respectively).

**Table 2:** Differences in the rate of neurologically favorable 1-M survival among the 3 bystander CPR groups before propensity score matching.

Subgroups	The rate of neurologically favorable 1-M survival, % (*N*)			Statistical analyses			
	No bystander CPR	Compression-only	Conventional	*P* by univariate analysis	Adjusted OR (95% CI) by multivariable logistic regression*		
					No bystander CPR	Compression-only	Conventional
All	2.4	3.2	6.6	*P* < 0.01	Reference	1.58 (0.94—2.67)	3.23 (1.82—5.72)
	(24/984)	(46/1428)	(31/470)		-	Reference	2.04 (1.25—3.35)
Medical	1.6	2.5	3.7	*P* = 0.09	Reference	2.64 (1.21—5.78)	3.23 (1.31—7.94)
	(11/709)	(27/1,095)	(13/349)		-	Reference	1.22 (0.59—2.52)
Non-medical	4.7	5.7	14.9	*P* < 0.01	Reference	1.21 (0.58—2.53)	3.61 (1.68—7.75)
	(13/275)	(19/333)	(18/121)		-	Reference	2.97 (1.49—5.94)
Witnessed	4.9	12.0	12.4	*P* = 0.01	Reference	3.09 (1.51—6.33)	3.63 (1.57—8.39)
	(12/245)	(33/276)	(15/121)		-	Reference	1.17 (0.59—2.34)
Unwitnessed	1.6	1.1	4.6	*P* < 0.01	Reference	0.78 (0.35—1.75)	2.68 (1.23—5.86)
	(12/739)	(13/1,152)	(16/349)		-	Reference	3.43 (1.60—7.33)

* Bystander CPR group, arrest witness, medical cause, shockable initial rhythm, sex, and response time interval (between emergency call receipt and EMS contact with the patient) were included.

### Comparisons after propensity score matching

Propensity score matching analysis included 453 patients in the compression-only and conventional BCPR groups. Comparisons of bystander witnesses, family witnesses, men, age, location, cause of OHCA, initial rhythm, prehospital defibrillation, time factor, and ROSC are presented. In a multivariate logistic regression analysis of all OHCAs, the neurologically favorable 1-M survival rate in the conventional BCPR group was significantly higher than that in the compression-only BCPR group (1.98; 1.03–3.81). In the subgroup analyses, outcomes in the conventional BCPR group were significantly better than those in the compression-only BCPR group for nonmedical and unwitnessed OHCA cases (2.83; 1.09–7.32 and 3.42; 1.09–10.76, respectively). No differences in outcomes were detected between conventional BCPR and the compression-only BCPR in medical and witnessed OHCA cases (1.23; 0.49–3.10 and 1.23; 0.53–2.86, respectively) (Table 3). Similarly, the propensity score matching analysis of the no BCPR group and the conventional BCPR group was performed for each of the 323 patients. The comparisons of characteristics after the matching are presented. In the univariate analysis, the neurologically favorable 1-M survival rate in the conventional BCPR group was significantly higher than that in the no BCPR group (*P* = 0.04). Multivariate logistic regression analysis showed that the conventional BCPR group had a significantly higher neurologically favorable 1-M survival rate than the no BCPR group (adjusted OR: 95% CI, 2.33; 1.02–5.35), regardless of subgroups.

**Table 3:** Comparisons of neurologically favorable 1-M survival rate between compression-only and conventional bystander CPR after propensity score matching.

Subgroups	The rate of neurologically favorable 1-M survival, % (*N*)		Adjusted OR (95% CI) with compression-only as reference^a^
	Compression-only	Conventional	
All	3.8 (17/453)	6.2 (28/453)	1.98 (1.03–3.81)
Medical	3.0 (10/338)	3.7 (12/338)	1.23 (0.49–3.10)
Non-medical	6.1 (7/115)	13.9 (16/115)	2.83 (1.09–7.32)
Witnessed	11.0 (13/118)	12.2% (14/115)	1.23 (0.53–2.86)
Unwitnessed	1.2 (4/335)	4.1 (14/338)	3.42 (1.09–10.76)

^a^ By multivariable logistic regression including bystander CPR group, arrest witness, medical cause, shockable initial rhythm, sex, and response time interval (between emergency call receipt and EMS contact with the patient) were included.

## Discussion

Epidemiological features of OHCAs in preschool children and outcome-associated factors were identified using a nationwide database from Japan. During the study period, 3,709 OHCAs occurred in preschool children (≤6 years old), or 14.2 OHCAs per 100,000 preschool children per year. In all preschool pediatric OHCA cases that were analyzed, the neurologically favorable 1-M survival rate was 3.5% (101/2,882). Our results show that preschool-aged OHCA patients had much poorer neurological outcomes than schoolchildren-aged OHCA patients (6–18 years old) in Japan (18, 19). In this study, we investigated the factors that affect the neurologically favorable 1-M survival rate in preschool pediatric OHCA patients. We focused on preschool pediatric OHCA cases, over half of which included infants, and limited the analysis to BCPR, yielding meaningful results.

In Japan, diagnosis of SIDS is made by the attending physicians through autopsies and mortality studies (20). SIDS falls into different categories when diagnosed without an autopsy, making it difficult to determine the exact incidence from the data in this study. SIDS, the leading cause of OHCAs in infants, occurs during sleep, especially in the prone or side position (21). However, apparent life-threatening events such as apnea, asphyxia, and cyanosis are not risk factors (22). In addition, hypoxemia due to asphyxiates precedes cardiac arrest and reduces brain function and gasps (23). Preschool children are less likely to complain of abnormal symptoms than adults and fall quietly into OHCA. Additionally, family members may be unaware of impending death signs in infants and children. OHCAs may occur at night, during sleep (including naps), or when the family members are occupied in household chores, leaving their child unsupervised. Blind spots and everyday noise may also hinder the discovery of OHCAs in pediatric patients. In this study, the inclusion of trauma and submersion associated with asphyxia events in OHCAs was done as these causes seem to be characteristic of preschool pediatric OHCAs, as found in previous reports (24).

The proportion of preschool pediatric OHCA cases receiving BCPR was approximately 66%, similar to the Korean study (24) conducted on the same age group, and higher than that in previous all-pediatric OHCA studies (5, 10, 25). The high BCPR rate in preschool pediatric OHCA patients may be because most bystanders are family members. However, compression-only BCPR accounted for three-quarters of these BCPR cases, and the bystanders tended not to opt for conventional BCPR. The proportion of conventional BCPR in this study was lower than that reported in previous all-pediatric OHCA studies (6, 23, 25). For this reason, in the American Heart Association CPR guidelines of 2010 and later (1, 2), pediatric (≥1 year) CPR is delivered according to the adult basic life support algorithm and emphasizes the importance of chest compressions. However, the European Resuscitation Council CPR guidelines point out that ventilation is as important as chest compressions in pediatric OHCAs up to puberty (4). In addition, CPR for infants is not emphasized because the general public rarely encounters cardiac arrest in infants. Mouth-to-mouth-and-nose rescue breathing in infants and children and chest compressions in infants are difficult to remember and to complete (26). It is possible that conventional CPR was not performed because bystanders lacked the knowledge of CPR procedures for infants and children. Consequently, intensive education is needed, especially for family members, about pediatric OHCAs and proper resuscitation procedures and measures.

In this study, before and after propensity score matching, conventional BCPR was associated with a higher neurologically favorable 1-M survival rate than compression-only BCPR. In addition, subgroup analyses after propensity score matching showed that in nonmedical and unwitnessed OHCA cases, neurological outcomes in the conventional BCPR group were significantly better than those in the compression-only BCPR group. Hypoxic-ischemic brain injury after cardiac arrest is the primary cause of poor mortality and neurological prognosis (27). Correction of hypoxemia and regional cerebral oxygen saturation by early ventilation during resuscitation has been shown to contribute to the achievement of ROSC and improvement of brain function prognosis (28). In this study, the ROSC rate was higher in conventional BCPR than in compression-only BCPR. In nonmedical causes, respiratory abnormalities due to trauma to the head or chest and rapid hypoxemia due to asphyxiation or drowning result in cardiac arrest; therefore, early ventilation is essential (2, 3, 29). During compression-only CPR, body-tored oxygen is distributed for up to 4 min after cardiac arrest (29). Considering that only one-fourth of the OHCAs in our study were witnessed, this should highlight concern for unwitnessed OHCA cases were thought to have already developed hypoxemia and early ventilation is required. In these two subgroups, oxygenation by early ventilation was adequate for subsequent brain resuscitation, which might have led to an improved neurologically favorable 1-M survival rate.

In this study, despite the high incidence of the medical cause of preschool pediatric OHCAs, only a few OHCA cases with shockable initial rhythm and prehospital defibrillation occurred, similar to that in a previous report (9). This finding of relatively few shockable initial rhythm events would also be consistent with a previous report stating that respiratory etiologies are more common than cardiac etiologies. However, the majority of OHCAs were unwitnessed, and it is possible that the shockable initial rhythm immediately after cardiac arrest changed to another ECG rhythm upon EMS arrival.

Conventional BCPR was not found to be superior to compression-only BCPR in OHCA cases due to medical causes probably because oxygen deficiency in medical OHCAs is less severe than that in non-medical OHCA, including submersion and asphyxia. Furthermore, tissue hypoxia progresses after cardiac arrest in medical OHCAs.

Without training in pediatric CPR, a bystander cannot perform CPR properly and has limited guidance from emergency medical dispatches. In this study, one-third of preschool pediatric OHCAs did not undergo BCPR, and a quarter of those who did, underwent conventional BCPR. Despite great interest in pediatric CPR in Japan, the lack of opportunities for CPR training may be another reason for the low rate of BCRP. Thus, focusing on educating family members living with preschool children and childcare facility staff about the importance of pediatric CPR, and increasing awareness about the risk of cardiac arrest occurring outdoors and at home, is warranted.

## Limitations

This study examined preschool pediatric OHCA patients, about half of whom were under one year of age. Although preschool pediatric OHCA is characterized differently from all-pediatric OHCA, a direct comparison with previous all-pediatric OHCA was not done due to the disparate age ranges. In addition, similar studies focusing on OHCA in preschool children are scarce and should be further investigated worldwide. EMS personnel interviewed the bystanders for factors associated with OHCA and also performed resuscitative efforts; however, bystander reports before EMS contact with the patient may be inaccurate. This study did not investigate the amount of administered epinephrine doses and the time from the onset of OHCA to the initiation of resuscitation; therefore, the neurological outcomes may have been affected. EMS reaction time, experience with CPR training, social status, and educational level, and other extrinsic factors may influence the relationship between the types of BCPR and a favorable neurological prognosis. Finally, the current study concluded at the end of 2019, effectively outside of the window of COVID-19 impact (30, 31). Therefore, we were not able to consider or compare the potential effects of current CPR standards on OHCA outcomes. This may be an area for further research.

## Conclusions

Bystanders, primarily families, rarely perform conventional BCPR for preschool pediatric OHCA patients. However, conventional BCPR increases the neurologically favorable outcomes of nonmedical and unwitnessed OHCAs. In pediatric OHCAs, which are often associated with a respiratory etiology, hypoxic-ischemic brain injury after cardiac arrest contributes to poor mortality and neurologic prognosis.

## Data Availability

The data analyzed in this study is subject to the following restrictions imposed by the Japanese Fire and Disaster Management Agency (FDMA). Requests to access these datasets should be directed to Fire and Disaster Management Agency 1-2 Kasumigaseki 2-chome, Chiyoda-ku. Tokyo 100-8926, Japan. Phone: +81-3-5253-5111 https://www.fdma.go.jp/en/post1.html.
